# Polyunsaturated fatty acid intake and prevalence of eczema and rhinoconjunctivitis in Japanese children: The Ryukyus Child Health Study

**DOI:** 10.1186/1471-2458-11-358

**Published:** 2011-05-21

**Authors:** Yoshihiro Miyake, Keiko Tanaka, Satoshi Sasaki, Masashi Arakawa

**Affiliations:** 1 Department of Preventive Medicine and Public Health, Faculty of Medicine, Fukuoka University, Fukuoka, Japan; 2 Department of Social and Preventive Epidemiology, School of Public Health, The University of Tokyo, Tokyo, Japan; 3 Field Science for Health and Recreation, Faculty of Tourism Sciences and Industrial Management, University of the Ryukyus, Okinawa, Japan

## Abstract

**Background:**

The recent increase in the prevalence of allergic disorders might be a consequence of increased intake of n-6 polyunsaturated fatty acids (PUFAs) and reduced intake of n-3 PUFAs. The current cross-sectional study examined the association between intake levels and the prevalence of eczema and rhinoconjunctivitis in Japanese children.

**Methods:**

Subjects were 23,388 schoolchildren aged 6-15 years residing in Okinawa. The presence of eczema and/or rhinoconjunctivitis was determined according to the criteria of the International Study of Asthma and Allergies in Childhood. A brief diet history questionnaire for children and adolescents was administered to acquire information on dietary factors. Adjustment was made for age, sex, residential municipality, number of siblings, smoking in the household, body mass index, paternal and maternal history of allergic diseases, and paternal and maternal educational level.

**Results:**

The prevalences of eczema and rhinoconjunctivitis in the previous 12 months were 7.0% and 8.0%, respectively. Consumption of PUFAs, n-3 PUFAs, α-linolenic acid, n-6 PUFAs, and linoleic acid was positively associated with the prevalence of eczema: the adjusted odds ratios (ORs) between extreme quintiles (95% confidence intervals [CIs], *P *for trend) were 1.26 (1.07-1.48, 0.04), 1.31 (1.11-1.54, 0.009), 1.31 (1.12-1.55, 0.003), 1.26 (1.07-1.48, 0.01), and 1.27 (1.08-1.49, 0.01), respectively. Arachidonic acid intake was independently inversely related to eczema: the adjusted OR between extreme quintiles was 0.81 (0.69-0.95, 0.0008). Eczema was not associated with eicosapentaenoic or docosahexaenoic acid intake, or with the ratio of n-3 to n-6 PUFA intake. Only arachidonic acid intake was statistically significantly related to the prevalence of rhinoconjunctivitis, showing a clear inverse linear trend: the adjusted OR between extreme quintiles was 0.86 (0.74-0.997, 0.03).

**Conclusions:**

Consumption of n-3 and n-6 PUFAs, especially α-linolenic acid and linoleic acid, may be positively associated with eczema. Arachidonic acid intake may be inversely related to eczema and rhinoconjunctivitis.

## Background

The prevalences of eczema and rhinoconjunctivitis have increased rapidly over the last few decades all over the world [1]. One study of Japanese elementary schoolchildren showed that the lifetime prevalence of eczema increased from 15.3% in 1985 to 24.2% in 1993 before decreasing to 16.5% in 2006, while the prevalence of rhinitis increased from 11.6% in 1983 to 24.7% in 2006 [2].

The recent increase in the prevalence of allergic disorders has been hypothesized to be the consequence of increased intake of n-6 polyunsaturated fatty acids (PUFAs) and reduced intake of n-3 PUFAs [3]. However, most of the available epidemiological information on the relationship between PUFA intake and atopic eczema or allergic rhinitis has come from studies of adults. In a study of Norwegian adults, females with moderate to severe atopic eczema had significantly lower intakes of eicosapentaenoic and docosahexaenoic acids compared to a reference group [4]. A significant inverse association was found between eicosapentaenoic acid intake and the risk of hay fever in a case-control study among German adults [5]. In a second cross-sectional study of German adults, a high ratio of n-6 to n-3 PUFA intake was significantly positively related to the prevalence of atopic eczema, but not hay fever, in females [6]. The results of a third cross-sectional study of German adults showed that a higher intake of α-linolenic acid, but not linoleic acid, arachidonic acid, or eicosapentaenoic acid, was significantly associated with a lower prevalence of allergic rhinitis [7]. Significant inverse exposure-response relationships between docosahexaenoic acid intake and the prevalence of atopic eczema and allergic rhinitis were found in pregnant Japanese women [8,9].

Turning to the epidemiological evidence concerning children, a cohort study showed that n-3 and n-6 PUFA consumption at 18 months and 3 years was not related to the risk of eczema in Australian children at the age of 5 years [10]. In 3 cohort studies among children in Norway and Sweden, fish intake was significantly inversely related to the risk of eczema and/or allergic rhinitis [11-13], yet no significant relationship was observed between fish consumption and eczema or allergic rhinitis among children in either a case-control study in Finland or a cross-sectional study in Italy [14,15]. Intake of margarine, which contains high levels of n-6 PUFAs, was positively associated with eczema in German children in a cohort study [16] and with allergic rhinitis in 2 cross-sectional studies of German children [17,18]. In the above-mentioned Finnish study, a positive association was shown between margarine intake and eczema but not between margarine intake and allergic rhinitis [14]. The present cross-sectional study examined the relationship between intake of specific types of PUFAs and the prevalence of eczema and rhinoconjunctivitis in Japanese children using data from the Ryukyus Child Health Study (RYUCHS).

## Methods

### Study population

Subjects of the RYUCHS were schoolchildren in 2 of the 41 municipalities in Okinawa Prefecture. Okinawa Prefecture is an island in the southernmost area of Japan, with a subtropical climate and a total population of almost 1,385,000. Naha City, the largest city in Okinawa Prefecture, with a total population of almost 315,000, is located in the southern region of the island; Nago City, with a total population of almost 60,000, is located in the center of the island. All 35 public elementary schools and 17 junior high schools in Naha City and all 17 public elementary schools and 8 junior high schools in Nago City participated in the RYUCHS between September 2004 and January 2005. The purpose of the RYUCHS, which was a cross-sectional survey, was to investigate the associations between various selected factors and health problems in children. A set of 2 self-administered questionnaires was distributed by teachers to all 38,212 schoolchildren aged 6-15 years. These questionnaires were completed at home by parents in the case of elementary schoolchildren and by students themselves and/or parents in the case of junior high school students. When research technicians detected missing answers or illogical data, the teachers returned the questionnaires to the parents for correction or completion. Finally, 28,885 sets of questionnaires (75.6%) were returned. Excluded from analysis were 5348 children whose questionnaires contained missing or illogical data on the variables under study. We further excluded 149 children with extremely low or high energy intake (< 2092 kJ/day or > 16,736 kJ/day) because their estimated dietary intake was likely to be unreliable. The final analysis included 23,388 children (61.2%). The RYUCHS was approved by the ethics committee of the Faculty of Medicine, Fukuoka University. Written informed consent was obtained from all subjects; in this survey, the signature of each child/adolescent on the questionnaires was considered informed consent made by both the child/adolescent and the parent(s)/caregiver(s).

### Measurements

Dietary habits during the preceding month were assessed using a brief self-administered diet history questionnaire (BDHQ) for Japanese children and adolescents [19], which was developed based on comprehensive (16 pages) [20,21] and brief (4 pages) versions of a validated self-administered diet history questionnaire for adults. Estimates of daily intake of foods (58 items in total), energy, and selected nutrients were calculated using an ad hoc computer algorithm for the BDHQ, based on the Standard Tables of Food Composition in Japan [22,23]. The use of dietary supplements, which is rare in Japan (8% of the general population) [24], was not incorporated into the analysis. Energy intake was adjusted by the residual approach for the analyses. The validity of the BDHQ for children and adolescents using dietary biomarkers (erythrocyte fatty acids and serum carotenoids) as the gold standard has been published elsewhere; briefly, Spearman correlation coefficients in 98 boys and 84 girls aged 13-14 years were 0.35 and 0.25 for eicosapentaenoic acid, 0.22 and 0.43 for docosahexaenoic acid, 0.21 and 0.13 for α-carotene, 0.23 and 0.33 for β-carotene, and 0.22 and 0.30 for β-cryptoxanthin, respectively [19]. The BDHQ also elicited information on the child's weight and height. Body mass index was calculated by dividing self-reported body weight (kg) by the square of self-reported height (m^2^).

The second questionnaire included questions on symptoms of eczema and rhinoconjunctivitis in the past 12 months based on the International Study of Asthma and Allergies in Childhood phase-I questionnaire [25,26]. Affirmative answers to the following three questions were required to confirm the presence of eczema: "Have you (Has your child) ever had an itchy rash which was coming and going for at least 6 months?", "Have you (Has your child) had this itchy rash at any time in the last 12 months?" and "Has this itchy rash at any time affected any of the following places: the folds of the elbows, behind the knees, in front of the ankles, under the buttocks, or around the neck, ears, or eyes?". Rhinoconjunctivitis was defined as present in the case of positive responses to the following two questions: "In the last 12 months, have you (has your child) had a problem with sneezing or a runny or blocked nose when you (he/she) did not have a cold or flu?" and "In the last 12 months, has this nose problem been accompanied by itchy/watery eyes?". The questionnaire also elicited information on age, sex, number of siblings, smoking in the household, paternal and maternal history of asthma, atopic eczema, and allergic rhinitis, and paternal and maternal educational level. A paternal or maternal history of asthma, atopic eczema, and allergic rhinitis was considered positive if the respective parent had experienced any self-reported symptoms of these allergic disorders since birth.

### Statistical analysis

Intake of selected nutrients was categorized at quintile points on the basis of the distribution of all study subjects. Age, sex, residential municipality, number of siblings, smoking in the household, body mass index, paternal and maternal history of allergic diseases, and paternal and maternal educational level were selected *a priori *as potential confounding factors. Age was classified into 3 categories (6-8, 9-11, and 12-15 years), number of siblings into 4 (0, 1, 2, and 3+), smoking in the household into 3 (never, former, and current), and paternal and maternal educational level into 4 (junior high school, high school, junior college or vocational technical school, and university). Body mass index was used as a continuous variable. Logistic regression analysis was applied to estimate crude odds ratios (ORs) and 95% confidence intervals (CIs) of eczema and rhinoconjunctivitis according to the quintile of dietary factors under study, with the lowest quintile as the reference. Multiple logistic regression analysis was used to control for potential confounding factors. Trend of association was assessed by a logistic regression model assigning consecutive integers to the quintiles of the exposure variables. Two-sided *P*-values less than 0.05 were regarded as statistically significant. All computations were performed using the SAS software package, version 9.1 (SAS Institute, Inc., Cary, NC, USA).

## Results

Data were incomplete for 5497 participants who were therefore subsequently excluded. Nevertheless, these excluded participants did provide information that allowed us to determine some differences between them and the participants who were included. Compared with the 5497 excluded subjects, the 23,388 included subjects were less likely to have no siblings, former smokers in the household, mothers who were less than 28 years of age at the time of the child's birth, and mothers with a history of asthma and atopic eczema, and were more likely to be young and to have a personal history of eczema and both fathers and mothers with high educational levels. There was no statistically significant difference between the excluded participants and study subjects with regard to the prevalence of rhinoconjunctivitis, sex, paternal history of asthma, atopic eczema, and allergic rhinitis, or maternal history of allergic rhinitis. It should be noted, however, that the total of 5497 excluded participants includes 149 participants who were excluded due to extremely low or high reported energy intake; when these 149 were excluded from the excluded participants, study subjects were more likely to have a high intake of n-3 and n-6 PUFAs in comparison to the remaining 5348 excluded participants.

Among the 23,388 included subjects, the prevalences of eczema and rhinoconjunctivitis in the previous 12 months were 7.0% and 8.0%, respectively. Table 1 shows the distribution of selected variables. About half of the subjects were exposed to household smoking at the time of the survey. Mean daily total energy intake and energy-adjusted consumption of n-3 and n-6 PUFAs were 8358.9 kJ and 2.0 g and 10.2 g, respectively.

**Table 1:** Distribution of selected characteristics in 23,388 schoolchildren, Ryukyus Child Health Study, Japan

Variable	No. (%) or mean (SD)
Age (% years)	
6-8	7063 (30.2)
9-11	8467 (36.2)
12-15	7858 (33.6)
Male sex (%)	11,509 (49.2)
Municipality (%)	
Naha City	19,206 (82.1)
Nago City	4182 (17.9)
Siblings (%)	
0	2144 (9.2)
1	7864 (33.6)
2	8823 (37.7)
3+	4557 (19.5)
Smoking in household (%)	
Never	10,083 (43.1)
Former	2381 (10.2)
Current	10,924 (46.7)
Paternal history of allergic diseases (%)^1^	5495 (23.5)
Maternal history of allergic diseases (%)^1^	6612 (28.3)
Paternal educational level (%)	
Junior high school	1813 (7.8)
High school	10,394 (44.4)
Junior college or vocational technical school	3484 (14.9)
University	7697 (32.9)
Maternal educational level (%)	
Junior high school	1173 (5.0)
High school	10,330 (44.2)
Junior college or vocational technical school	9788 (41.9)
University	2097 (9.0)
Body mass index (kg/m^2^)	17.8 (3.1)
Daily nutrient intake^2^	
Total Energy (kJ)	8358.9 (2253.2)
Polyunsaturated fatty acids (g)	14.3 (3.1)
n-3 Polyunsaturated fatty acids (g)	2.0 (0.6)
α-Linolenic acid (18:3 n-3) (g)	1.6 (0.5)
Eicosapentaenoic acid (20:5 n-3) (g)	0.11 (0.09)
Docosahexaenoic acid (22:6 n-3) (g)	0.27 (0.15)
n-6 Polyunsaturated fatty acids (g)	10.2 (2.7)
Linoleic acid (18:2 n-6) (g)	10.0 (2.7)
Arachidonic acid (20:4 n-6) (g)	0.12 (0.04)

^1 ^Asthma, atopic eczema, or allergic rhinitis.

^2 ^Nutrient intake was adjusted for total energy intake using the residual method.

ORs and 95% CIs for the prevalence of eczema and rhinoconjunctivitis according to dietary intake of specific types of PUFAs are shown in Table 2. Compared with PUFA intake in the first quintile, the intake levels in the third and fifth quintiles were significantly associated with an increased prevalence of eczema. After adjustment for confounders under study, the positive associations remained significant, albeit slightly attenuated: the adjusted OR between extreme quintiles was 1.26 (95% CI: 1.07-1.48, *P *for trend = 0.04). Higher intakes of n-3 PUFAs and α-linolenic acid were independently positively related to the prevalence of eczema: the adjusted ORs between extreme quintiles were 1.31 (95% CI: 1.11-1.54, *P *for trend = 0.009) and 1.31 (95% CI: 1.12-1.55, *P *for trend = 0.003), respectively. Significant positive relationships were also observed between intake levels of n-6 PUFAs and linoleic acid and the prevalence of eczema: the adjusted ORs between extreme quintiles were 1.26 (95% CI: 1.07-1.48, *P *for trend = 0.01) and 1.27 (95% CI: 1.08-1.49, *P *for trend = 0.01), respectively. On the other hand, higher intake of arachidonic acid was independently related to a reduced prevalence of eczema: the adjusted OR between extreme quintiles was 0.81 (95% CI: 0.69-0.95, *P *for trend = 0.0008). Such an inverse association was also detected for rhinoconjunctivitis: the adjusted OR between extreme quintiles was 0.86 (95% CI: 0.74-0.997, *P *for trend = 0.03). Intakes of eicosapentaenoic acid and docosahexaenoic acid and the ratio of n-3 to n-6 PUFA intake were not significantly related to the prevalence of eczema. No significant exposure-response associations were found between intakes of PUFAs, n-3 PUFAs, α-linolenic acid, eicosapentaenoic acid, docosahexaenoic acid, n-6 PUFAs, and linoleic acid and the ratio of n-3 to n-6 PUFA intake and the prevalence of rhinoconjunctivitis.

**Table 2:** Odds ratios (ORs) and 95% confidence intervals (CIs) for eczema and rhinoconjunctivitis in relation to dietary intake of polyunsaturated fatty acids in 23,388 schoolchildren, Ryukyus Child Health Study, Japan

	Eczema			Rhinoconjunctivitis		
		
**Variable**^**1**^	Prevalence (%)	Crude OR (95% CI)	**Adjusted OR (95% CI)**^**2**^	Prevalence (%)	Crude OR (95% CI)	**Adjusted OR (95% CI)**^**2**^
Polyunsaturated fatty acids						
Q1 (10.7)	6.1	1.00	1.00	8.8	1.00	1.00
Q2 (12.8)	6.8	1.13 (0.96-1.34)	1.10 (0.94-1.31)	7.6	0.86 (0.74-0.99)	0.86 (0.74-0.999)
Q3 (14.2)	7.7	1.29 (1.10-1.51)	1.23 (1.05-1.45)	7.2	0.81 (0.69-0.94)	0.81 (0.70-0.95)
Q4 (15.6)	6.5	1.08 (0.92-1.28)	1.02 (0.86-1.21)	7.9	0.89 (0.77-1.03)	0.89 (0.77-1.04)
Q5 (18.0)	7.9	1.33 (1.13-1.56)	1.26 (1.07-1.48)	8.6	0.98 (0.85-1.13)	0.97 (0.83-1.12)
*P *for trend		0.005	0.04		0.96	0.83
n-3 Polyunsaturated fatty acids						
Q1 (1.3)	5.8	1.00	1.00	8.1	1.00	1.00
Q2 (1.7)	6.9	1.20 (1.02-1.42)	1.16 (0.98-1.37)	8.2	1.01 (0.87-1.17)	1.01 (0.87-1.17)
Q3 (2.0)	7.4	1.30 (1.10-1.53)	1.22 (1.04-1.45)	7.1	0.87 (0.75-1.01)	0.87 (0.74-1.01)
Q4 (2.3)	6.9	1.21 (1.02-1.42)	1.12 (0.94-1.32)	8.1	1.00 (0.86-1.16)	0.99 (0.85-1.15)
Q5 (2.8)	7.9	1.40 (1.19-1.64)	1.31 (1.11-1.54)	8.6	1.07 (0.93-1.24)	1.05 (0.90-1.22)
*P *for trend		0.0003	0.009		0.45	0.63
α-Linolenic acid (18:3 n-3)						
Q1 (1.0)	5.8	1.00	1.00	8.6	1.00	1.00
Q2 (1.3)	6.7	1.17 (0.99-1.39)	1.14 (0.96-1.35)	8.0	0.93 (0.80-1.08)	0.93 (0.80-1.08)
Q3 (1.5)	7.5	1.32 (1.12-1.56)	1.25 (1.06-1.47)	7.2	0.82 (0.71-0.95)	0.82 (0.71-0.96)
Q4 (1.8)	7.1	1.24 (1.05-1.46)	1.15 (0.97-1.36)	8.1	0.94 (0.81-1.09)	0.94 (0.81-1.09)
Q5 (2.2)	8.0	1. 41 (1.20-1.66)	1.31 (1.12-1.55)	8.2	0.95 (0.82-1.10)	0.93 (0.80-1.08)
*P *for trend		< 0.0001	0.003		0.57	0.41
Eicosapentaenoic acid (20:5 n-3)						
Q1 (0.03)	6.4	1.00	1.00	8.3	1.00	1.00
Q2 (0.07)	6.8	1.06 (0.90-1.25)	1.04 (0.88-1.23)	7.8	0.94 (0.81-1.10)	0.95 (0.82-1.11)
Q3 (0.09)	7.1	1.10 (0.94-1.30)	1.07 (0.91-1.26)	7.6	0.92 (0.79-1.06)	0.91 (0.78-1.06)
Q4 (0.12)	7.5	1.18 (1.002-1.38)	1.15 (0.98-1.35)	7.5	0.90 (0.78-1.05)	0.90 (0.77-1.05)
Q5 (0.21)	7.2	1.13 (0.96-1.33)	1.12 (0.95-1.31)	8.9	1.09 (0.94-1.26)	1.10 (0.95-1.27)
*P *for trend		0.06	0.09		0.41	0.42
Docosahexaenoic acid (22:6 n-3)						
Q1 (0.13)	6.9	1.00	1.00	8.5	1.00	1.00
Q2 (0.20)	6.6	0.95 (0.81-1.12)	0.94 (0.80-1.11)	7.6	0. 89 (0.76-1.03)	0. 90 (0.77-1.05)
Q3 (0.24)	7.5	1.08 (0.93-1.27)	1.04 (0.88-1.21)	7.6	0.88 (0.76-1.02)	0.86 (0.74-0.998)
Q4 (0.30)	7.0	1.01 (0.86-1.18)	0.99 (0.84-1.16)	7.9	0.92 (0.79-1.06)	0.92 (0.79-1.06)
Q5 (0.43)	7.0	1.01 (0.86-1.19)	1.01 (0.86-1.18)	8.5	1.00 (0.87-1.16)	1.00 (0.86-1.16)
*P *for trend		0.67	0.75		0.85	0.92
n-6 Polyunsaturated fatty acids						
Q1 (7.1)	6.1	1.00	1.00	8.6	1.00	1.00
Q2 (8.8)	6.8	1.12 (0.95-1.32)	1.07 (0.91-1.27)	7.9	0.91 (0.78-1.05)	0.91 (0.78-1.05)
Q3 (10.1)	7.2	1.19 (1.01-1.40)	1.14 (0.96-1.34)	7.7	0.88 (0.76-1.02)	0.90 (0.77-1.05)
Q4 (11.3)	7.0	1.15 (0.97-1.35)	1.08 (0.91-1.27)	7.5	0.86 (0.74-1.001)	0.87 (0.75-1.02)
Q5 (13.5)	8.0	1.33 (1.13-1.56)	1.26 (1.07-1.48)	8.5	0.99 (0.86-1.15)	0.98 (0.85-1.14)
*P *for trend		0.001	0.01		0.71	0.68
Linoleic acid (18:2 n-6)						
Q1 (6.9)	6.1	1.00	1.00	8.6	1.00	1.00
Q2 (8.7)	6.8	1.12 (0.95-1.32)	1.08 (0.91-1.27)	7.9	0.91 (0.78-1.05)	0.91 (0.79-1.06)
Q3 (9.9)	7.2	1.20 (1.02-1.41)	1.14 (0.97-1.34)	7.5	0.86 (0.74-1.002)	0.88 (0.76-1.03)
Q4 (11.1)	6.9	1.13 (0.96-1.33)	1.06 (0.90-1.26)	7.5	0.86 (0.74-0.999)	0.87 (0.75-1.02)
Q5 (13.3)	8.0	1.34 (1.14-1.57)	1.27 (1.08-1.49)	8.6	1.00 (0.87-1.16)	1.00 (0.86-1.15)
*P *for trend		0.001	0.01		0.77	0.76
Arachidonic acid (20:4 n-6)						
Q1 (0.07)	7.9	1.00	1.00	8.9	1.00	1.00
Q2 (0.10)	7.5	0.95 (0.81-1.10)	0.92 (0.78-1.07)	7.8	0.87 (0.75-1.01)	0.88 (0.76-1.02)
Q3 (0.12)	6.7	0.83 (0.71-0.98)	0.80 (0.68-0.94)	8.2	0.92 (0.79-1.06)	0.91 (0.78-1.05)
Q4 (0.14)	6.3	0.79 (0.67-0.92)	0.76 (0.65-0.89)	7.5	0.84 (0.72-0.97)	0.83 (0.72-0.97)
Q5 (0.17)	6.6	0.83 (0.71-0.97)	0.81 (0.69-0.95)	7.8	0.87 (0.75-1.01)	0.86 (0.74-0.997)
*P *for trend		0.002	0.0008		0.05	0.03
n-3/n-6 Polyunsaturated fatty acid ratio						
Q1 (0.17)	6.0	1.00	1.00	7.4	1.00	1.00
Q2 (0.18)	7.1	1.20 (1.02-1.41)	1.17 (0.99-1.38)	7.8	1.06 (0.91-1.23)	1.05 (0.90-1.23)
Q3 (0.20)	7.0	1.18 (0.997-1.39)	1.14 (0.96-1.34)	8.1	1.10 (0.94-1.28)	1.07 (0.92-1.25)
Q4 (0.21)	7.6	1.28 (1.09-1.51)	1.21 (1.03-1.42)	8.0	1.08 (0.93-1.26)	1.04 (0.89-1.21)
Q5 (0.23)	7.2	1.21 (1.03-1.43)	1.17 (0.99-1.38)	8.7	1.19 (1.03-1.38)	1.16 (0.99-1.35)
*P *for trend		0.02	0.07		0.03	0.11

^1 ^Quintile medians in g/day adjusted energy intake using the residual method are given in parentheses, except for the ratio of n-3 to n-6 polyunsaturated fatty acids, which was based on crude intake in g/day.

^2 ^Adjustment for age, sex, residential municipality, number of siblings, smoking in the household, body mass index, paternal and maternal history of allergic diseases, and paternal and maternal educational level.

## Discussion

The current results show that higher consumption levels of PUFAs, n-3 PUFAs, α-linolenic acid, n-6 PUFAs, and linoleic acid are independently associated with an increased prevalence of eczema whereas intake of arachidonic acid is independently inversely related to the prevalence of eczema. Consumption of eicosapentaenoic and docosahexaenoic acid and the ratio of n-3 to n-6 PUFA intake were not associated with eczema. Only arachidonic acid intake was statistically significantly related to the prevalence of rhinoconjunctivitis, showing a clear inverse linear trend. Our results are at variance with those of an Australian study that reported no associations between n-3 and n-6 PUFA intake and childhood eczema [10]. Partial agreement was noted with the results of studies that showed a positive association between margarine intake and eczema and no relationship between fish intake and allergic rhinitis [14-16], but our results are at variance with reports of a significant inverse relationship between fish intake and eczema and allergic rhinitis [11-13] and with reports of a significant positive association between margarine intake and allergic rhinitis [17,18]. Previously, using data from the RYUCHS, we reported that higher intake levels of PUFAs, n-3 PUFAs, n-6 PUFAs, and linoleic acid were significantly related to an increased prevalence of wheeze [27], though we found no associations between intakes of α-linolenic acid, eicosapentaenoic acid, docosahexaenoic acid, and arachidonic acid, or the ratio of n-3 to n-6 PUFA intake, and wheeze [27]. Exposure-response relationships were not found between the intake of any type of PUFA and the prevalence of asthma [27]. Those findings are in partial agreement with the current results. With regard to arachidonic acid, our results are in partial agreement with those from the KOALA Birth Cohort Study, which showed that the risk of eczema in the first 12 months of life significantly decreased with increasing levels of arachidonic acid in plasma phospholipids in pregnancy, although there was no relationship between the arachidonic acid levels and eczema later in life [28]. The current results are inconsistent with those of a cross-sectional study showing a positive association between concentrations of arachidonic acid in serum phospholipids and hay fever in German adults [29]. The direction of the relationship between arachidonic acid and allergic disorders might change according to the timing of the exposure: an inverse relationship may exist starting in utero and continuing through childhood, whereas a positive relationship may exist in adulthood.

Linoleic acid can be converted to arachidonic acid, which is usually the major substrate for eicosanoid synthesis [30]. Eicosanoids, which consist of prostaglandins, thromboxanes, leukotrienes, and other oxidized derivatives, are generated from arachidonic acid by cyclooxygenase and lipoxygenase enzymes [30]. Some eicosanoids are implicated in clinical manifestations of allergic disorders, although individual prostaglandins might augment or inhibit inflammation related to allergic reactions, depending upon their specific action [30,31]. One current paradigm is that prostaglandin D_2_, prostaglandin F_2α_, and thromboxane A_2 _increase allergic inflammation whereas prostaglandin E_2 _and prostaglandin I_2 _inhibit allergen-induced inflammatory responses [31]. Given the positive relationships between linoleic acid intake and the production of prostaglandin D_2_, prostaglandin F_2α_, and thromboxane A_2_, the current results showing an association of linoleic acid intake with an increased prevalence of eczema might be considered reasonable, although no relationship between linoleic acid intake and rhinoconjunctivitis was shown. On the other hand, higher arachidonic acid intake may have led to increased production of prostaglandin E_2 _and prostaglandin I_2_, with a consequent decrease in eczema and rhinoconjunctivitis in the subjects studied.

Long chain n-3 PUFAs inhibit arachidonic acid incorporation into cell membranes and inhibit arachidonic acid metabolism to eicosanoids [30]. Given that higher α-linolenic acid intake especially inhibits the production of prostaglandin E_2 _and prostaglandin I_2_, the present results concerning α-linolenic acid intake and eczema also might be reasonable although a null relationship between such intake and rhinoconjunctivitis was found.

In Japan, fish intake is high. The mean values for daily intake of eicosapentaenoic and docosahexaenoic acids in this population were 0.11 g and 0.27 g, respectively. In many parts of Europe, the daily intake of eicosapentaenoic acid + docosahexaenoic acid by adults is < 100 mg [32]. In a nested case-control study of Australian children that showed a significant positive association between the ratio of n-6 to n-3 PUFAs in the diet and the risk of asthma [33], the mean value of daily n-3 PUFA intake and the ratio of n-3 to n-6 PUFA intake among 169 controls were 1.05 g and 0.065, respectively; these values were much lower than the corresponding figures in this study (2.03 g and 0.199, respectively). Therefore, protective associations of n-3 PUFA intake and the ratio of n-3 to n-6 PUFA intake with eczema and rhinoconjunctivitis might be detected when consumption of n-3 PUFAs is very low. Alternatively, unrecognized active agents in fish might have counteracted the benefit of marine-origin n-3 PUFAs in eczema and rhinoconjunctivitis. For example, methylmercury and dioxins are accumulated in fish and shellfish through the marine food web. One study has found a significant correlation between fish consumption and hair mercury levels, reporting that hair mercury levels were much higher in Japanese women residing in Canada than in Canadian women [34].

Eggs, meat, fish, milk, and sweets are major sources of arachidonic acid intake among Japanese people [35]. Meat, milk, and sweets are also major sources of *trans *fatty acid intake among Japanese people [36]. Arachidonic acid intake may be to some extent correlated with *trans *fatty acid intake. In this study, data on *trans *fatty acid intake were not available because the Standard Tables of Food Composition in Japan do not include information on *trans *fatty acids. In the KOALA Birth Cohort Study, higher concentrations of *trans *fatty acids from a rumenic source in human breast milk were associated with a lower risk of eczema at 2 years of age [37]. In contrast, an ecological study using data from the International Study of Asthma and Allergies in Childhood showed positive relationships between *trans *fatty acid intake and the prevalences of asthma, eczema, and rhinoconjunctivitis [38].

The current study had methodological advantages. Study subjects were homogeneous in terms of age and geographical background. That the study included a large number of both subjects and confounders was likely to minimize problems due to small sample size, inadequate data on potential confounders, and reduced statistical power. However, residual confounding could not be ruled out. In particular, no adjustment was made for data on allergen exposure and the home environment. The core questions used to assess the outcomes under study had been validated by the International Study of Asthma and Allergies in Childhood phase-I study, but validation tests of the questions had not been performed in this population. Moreover, no attempt was made to ascertain outcome status through reviews of medical records.

Other weaknesses include the fact that dietary data were obtained through a self-administered dietary assessment questionnaire (i.e., BDHQ). Because actual dietary habits were not observed, the results should be interpreted with caution, although the validity of the BDHQ appears reasonable, as described above. The estimates of consumption of specific types of PUFAs derived from the BDHQ might not reflect long-term intake by subjects. Nevertheless, subjects with eczema or rhinoconjunctivitis were likely to be unaware of the possible ill effects of PUFA intake. The consequence would have been underestimation in our results. Information on dietary supplements was not available in the present study; their use, however, is uncommon in Japan [24]. The BDHQ was answered by parents in the case of elementary schoolchildren and by students and/or parents in the case of junior high school students. Dietary data on children and adolescents is, in the main, prone to reporting error [39]. We cannot predict whether possible selective misreporting of dietary intake would systematically deflate or inflate the estimates of these dietary variables. In any case, random misclassification in our study was likely, and it probably weakened the evidence for any true relationships. Information on self-reported body weight and height was not validated, and thus body mass index might have been biased.

Only 61.2% of the eligible subjects were included in the current analysis, suggesting that selection bias might have been inevitable. In fact, as mentioned above, there was a significant difference between the excluded participants and study subjects with regard to several factors.

Okinawa has unique characteristics regarding its geographical location, as it is located at the southern extremity of Japan, is close to Taiwan, and is the only prefecture in Japan situated in a subtropical climate zone. Moreover, Okinawa is less developed than the mainland because it only reverted to Japanese administration in 1972. The distribution of a variety of environmental factors including diet and socioeconomic status as well as allergy in Okinawa is likely to differ from that in the mainland of Japan. For example, according to the criteria of the International Study of Asthma and Allergies in Childhood, the prevalences of eczema and rhinoconjunctivitis in the previous 12 months were 14.5% and 23.9%, respectively, among Japanese adolescents in Suita City, an urban area in the mainland [40]. Because Japanese cedar pollinosis is a major public health problem in the mainland of Japan, this finding with regard to rhinoconjunctivitis is not unexpected. Because Okinawa has comparatively few Japanese cedars, however, people in Okinawa are not as likely to experience rhinoconjunctivitis. We should, therefore, be cautious in generalizing the present results to Japanese children in the mainland. Another limitation is that we could not take clustering of conditions within families into consideration, as no information was available on familial relationships among the subjects. This lack of data might influence relationships of interest.

## Conclusion

This is the first epidemiological study among children to report that consumption of n-3 and n-6 PUFAs, especially α-linolenic acid and linoleic acid, is significantly positively associated with the prevalence of eczema. Also, new evidence is presented showing significant inverse relationships between arachidonic acid intake and the prevalence of eczema and rhinoconjunctivitis. Because different mechanisms might be involved in the manifestation of eczema and that of rhinoconjunctivitis, there might also be differences in the detrimental effects of α-linolenic acid and linoleic acid. Owing to the cross-sectional nature of this study, which eliminates any causal inferences, and the incompleteness of the biological explanations for these associations, further investigations are needed to ascertain whether the relationships observed in this study are replicated in other populations, especially in populations with high n-3 PUFA intake.

## Competing interests

The authors declare that they have no competing interests.

## Authors' contributions

All authors contributed to the study concept and design and the acquisition of data. YM was responsible for the analysis and interpretation of data and the drafting of the manuscript. All authors participated in critically revising the manuscript and approved the final version of the manuscript.

## Pre-publication history

The pre-publication history for this paper can be accessed here:

http://www.biomedcentral.com/1471-2458/11/358/prepub
